# Climatic determinants of plant phenology in vernal pool habitats

**DOI:** 10.1002/ajb2.70064

**Published:** 2025-06-28

**Authors:** Brandon Thomas Hendrickson, Jenna Aubrie Benterou, Robert Martin, Jason P. Sexton

**Affiliations:** ^1^ Department of Biology University of Louisiana at Lafayette Lafayette LA USA; ^2^ Department of Life and Environmental Sciences University of California Merced CA USA

**Keywords:** citizen science, climate change, ephemeral wetlands, growing degree hours, phenology, specialist

## Abstract

**Premise:**

The floral phenology of vernal pool plants is little understood despite being a crucial developmental stage for producing seeds and determining population growth rates. Vernal pools are ephemerally aquatic habitats harboring species adapted to predictable seasonal fluctuations between desiccated and inundated conditions; thus, vernal pool plant phenology is predicted to be particularly responsive to interannual climate variability.

**Methods:**

For two vernal pool species, *Limnanthes douglasii* subsp. *rosea* (meadowfoam), a vernal pool specialist, and *Trifolium variegatum* (whitetip clover), a generalist vernal pool associate, we characterized flowering onset, termination, and duration in response to interannual variation of winter precipitation and growing degree hours (GDH). We recorded phenology over 7 years from 2016 to 2022 during a period of high climatic variability, which served as a robust data set.

**Results:**

Warmer and drier environmental conditions during early growth periods were strongly associated with advanced floral phenology later in the life cycle for both species. The floral duration of the vernal pool specialist was influenced by position along the inundation gradient, whereas no such pattern was observed for the vernal pool associate.

**Conclusions:**

To our knowledge, this is the first study quantifying the relationship between vernal pool floral phenology and climate, offering insights into how phenology may shift in response to modern climate change.

Vernal pool habitats, ephemeral wetlands, harbor many specialist plant species (Holland, 1976; Stone, 1990) adapted to sharp transitions from extreme wet to extreme dry conditions (Keeley and Zedler, 1998). Agricultural expansion and urban development have destroyed 85–95% of the original vernal pool habitat native to California (Holland, 1998, 2009; Witham et al., 2014). Consequently, many vernal pool organisms are endangered and continuously threatened by invasion and human encroachment, elevating the need for understanding the role climate change may play in further disrupting this already fragile habitat. Vernal pools annually fill with water during the winter and dry over the spring season, offering a unique system for investigating how climate variation in the winter and spring affect flowering onset, termination, and duration. However, neither the abiotic cues that trigger flowering nor the degree to which flowering onset, duration, and termination change in response to climate is currently understood in vernal pool habitats. We aimed to quantify the relationship between seasonal climate conditions and interannual phenological patterns of two vernal pool plant species (one specialized, the other more generalist) over a 7‐year study (2016–2022) to better understand the susceptibility of vernal pool habitats to present and future climate change. We conducted this research in a community science context, involving undergraduate student volunteers from the University of California, Merced, every spring in the collection of phenology data.

Vernal pools are shallow depressions underlain by an impermeable soil horizon (Holland and Jain, 1988; Boone et al., 2006), either clay or rock, that cyclically flood during the wet season, gradually dry throughout the spring, and then dry during the summer (Keeley and Zedler, 1998; Solomeshch et al., 2007). Few plant species can tolerate such extremes of water availability, and consequently, the flora is composed of many specialists (Hanes and Stromberg 1998; Keeley and Zedler, 1998) and other more widespread vernal pool associates growing along pool margins. The predictably sharp transitions from inundation to desiccation that characterize vernal pools can be divided into two biologically informative temporal phases: (1) the aquatic phase, when plants quickly germinate following the first rain and grow slowly as diminutive rosettes while submerged, and (2) the terrestrial phase, which commences as water recedes, and vernal pool plants rapidly complete their life cycle while soils are saturated (Keeley and Zedler, 1998; Pollak and Kan, 1998; Bauder, 2000; Gerhardt and Collinge, 2003). These phases are largely concentrated from October to May in the northern hemisphere (Zedler, 2003). Consequently, annual averages of precipitation and temperature are often uninformative of growth patterns of vernal pool plant communities (Javornik and Collinge, 2016); thus, studies of community composition and germination phenology have focused on late fall, winter, and early spring weather patterns. Much knowledge of vernal pool plant phenology has been acquired using this heuristic, such as correlating hydroperiod with seedling emergence (Bliss and Zedler, 1998; Collinge et al., 2013), community composition (Barbour et al., 2003; Deil, 2005; Solomeshch et al.,^2007^; Gosejohan et al., 2017), phylogenetic diversity (Hendrickson, 2024), and plant traits (Kraft et al., 2014). However, the influence of climatic variation, particularly during the aquatic and terrestrial phases, has not been assessed in the context of flowering phenology in natural vernal pools.

The onset of flowering acts as a fingerprint of climatic variation in an ecosystem (Parmesan and Yohe, 2003; Fatima et al., 2020; Menzel et al., 2020) that underlies reproductive potential (Miller‐Rushing et al., 2010) and provisioning services for pollinators (Rafferty and Ives, 2011). The floral phenology of many terrestrial wildflowers exhibits sensitivity to air temperature (Stuble et al., 2021), precipitation (Matthews and Mazer, 2016), and photoperiod (Friedman and Willis, 2013), whereas aquatic plants are responsive to inundation level (Greet et al., 2013; Calero et al., 2015) and water temperature (Calero et al., 2015). Furthermore, growing degree hours (GDH) has been commonly used to predict flowering in agricultural and tree species (Major, 1980; Rattigan and Hill, 1986; White, 1995; Leon et al., 2001), though GDH has seldom been applied to explain wildflower phenology (see Javornik and Collinge, 2016). Flowering onset of two vernal pool species grown in constructed mesocosms exhibited significant associations with inundation length (Collinge et al., 2013), suggesting a similar floral response as primarily aquatic species. Furthermore, prior studies of California vernal pool plants have shown important transitions from germination, survival, growth, and reproduction that parallel seasonal shifts of water volume (Keeley and Zedler, 1998; Collinge et al., 2013; Gosejohan et al., 2017). However, given that vernal pools are ephemerally aquatic, and many species grow and flower under mesic rather than flooded conditions, climatic factors such as air temperature and GDH are likely to also influence floral phenology. In addition, variation in seasonal precipitation may have a large effect on the phenology of semiaquatic species by influencing inundation length and degree of exposure to air temperature. The influence of climatic variation during the terrestrial phase of vernal pools has been understudied; thus, the impacts that air temperature and GDH have on flowering onset, termination, and duration are not currently understood in vernal pool habitats.

Vernal pool specialists and associates differ in several features that may influence phenological responses to climate variation. Vernal pool associates are found in vernal pool habitats but are not restricted exclusively to them and occupy a wide geographic and ecological range. This contrasts with vernal pool specialists, which are highly specialized to the unique hydrological cycles of vernal pools (Zedler, 2003; Keeley and Zedler, 1998; Holland and Jain, 1984; Bauder, 2005). Given that vernal pool specialists time their life cycle in response to seasonal patterns of inundation and desiccation, these plants may be highly sensitive to changes in temperature, precipitation, and the timing and duration of inundation (Zedler, 2003). In contrast, vernal pool associates tolerate a broader range of hydrological conditions, and thus their growth and, potentially, phenology are less influenced by the timing and duration of inundation (Keeley and Zedler, 1998). Consequently, vernal pool specialists may respond more dramatically to interannual climate variability, including increasing variability caused by contemporary climate change, than widespread vernal pool associates.

Climate change is manifesting in several ways, including increasing temperatures, drought, and weather variability (Dettinger and Cayan, 1994; Bauder, 2005; Brooks, 2009). Global patterns of shifting floral initiation in response to warming temperatures and lower precipitation have been recorded on six of the seven continents and in many biomes (Post et al., 2018; Hassan et al., 2023; Zhou et al., 2023). However, phenological studies have not yet included vernal pool habitats, which are important for understanding ecological patterns in ephemeral environments (e.g., Ruiz‐Ramos et al., 2023). The degree to which climate is changing depends on the season; fall minimum and maximum temperatures are rising more rapidly than spring temperatures compared to historical averages in the Central Valley of California (He et al., 2018). Given that vernal pool species mostly emerge in early winter and bloom in spring, it is important to investigate which season has a larger impact on flowering onset and termination. Additionally, there is currently no clear pattern of how flowering duration (the length of time between flowering onset and flowering termination) will change in response to warmer and drier conditions (Li et al., 2020; Puchalka, 2022). Understanding the phenological responses of vernal pool species to temperature and precipitation will provide valuable insight into how vernal pool communities will react to ongoing climate change.

We studied how floral phenology of one vernal pool specialist [meadowfoam, *Limnanthes douglassii* subsp. *rosea* (Benth.) C.T. Mason; Limnanthaceae] and one vernal pool associate (whitetip clover, *Trifolium variegatum* Nutt.; Fabaceae) respond to variation in temperature and precipitation in natural vernal pools. Both species germinate in the winter after the first rains and rapidly grow during the spring as temperatures rise and water recedes. However, vegetative growth proceeds throughout the inundation phase of the vernal pool that may contribute to flowering phenology in the spring. By dividing the growing period into two time bins (i.e., early winter and late winter), we examined the independent influence of climate in the early and the late winter on spring flowering phenology. We asked three questions: (1) What climatic factors best explain flowering onset, flowering termination, and flowering duration? (2) Is the flowering phenology of the vernal pool specialist and associate responding to temperature and precipitation similarly? (3) How does floral duration change in response to climatic variation and pool topography?

## MATERIALS AND METHODS

### Study system

**Figure 1:**
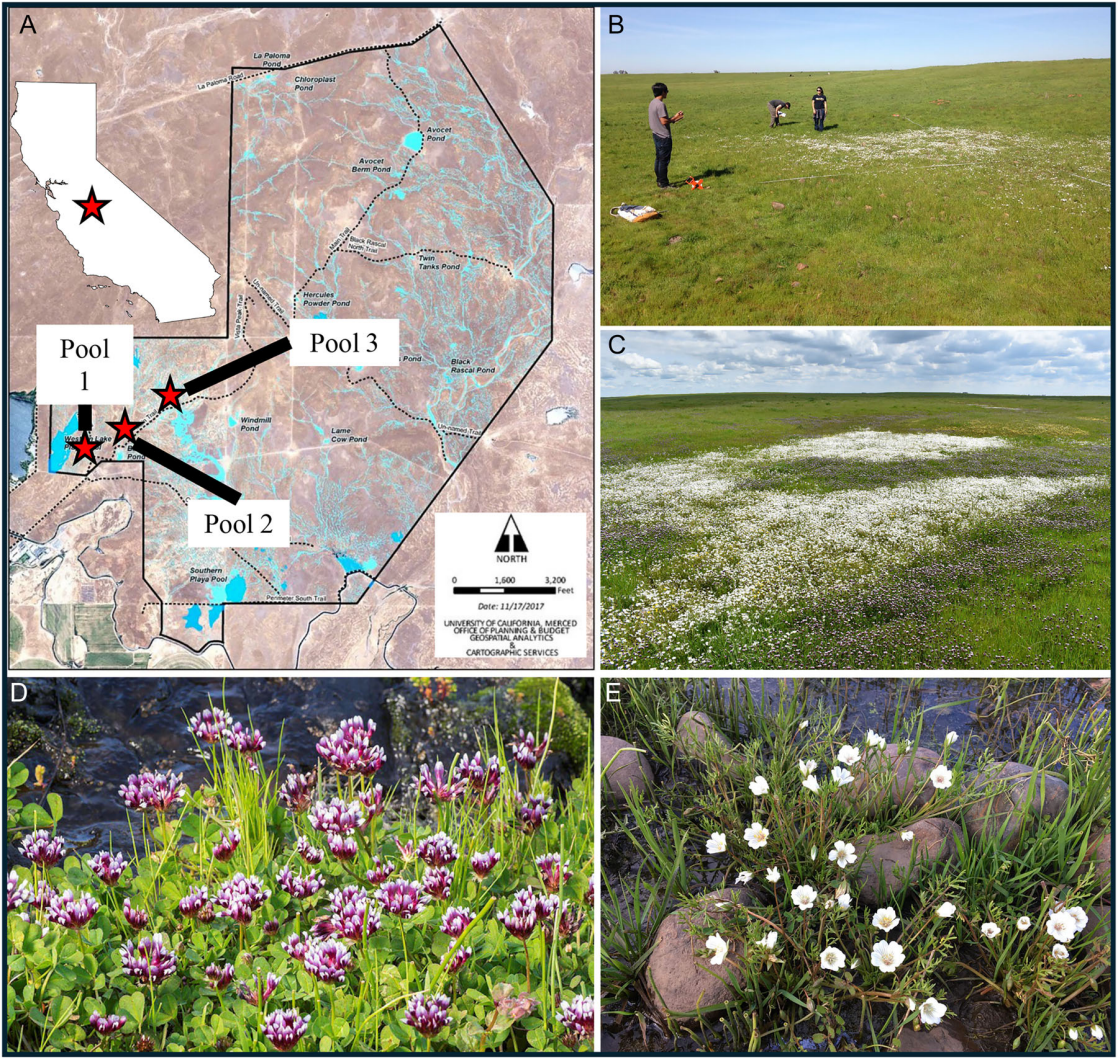
(A) Map of the Merced Vernal Pool and Grassland Reserve (MVPGR) with the locations of three observation pools (red stars) investigated from 2016 to 2022. The Merced Vernal Pool Megacomplex is denoted by a red star in the white map of California. (B) Undergraduate citizen scientists recording the density of plants, flowers, and seeds of meadowfoam and whitetip clover. (C) A vernal pool during peak bloom with white meadowfoam flowers in the center and whitetip clover along the edges. (D, E) Photos of the two focal species: (D) whitetip clover (*Trifolium variegatum)* and (E) meadowfoam (*Limnanthes douglasii* subsp. *rosea*). Photography credits: (B, E) Jason Sexton, (C) Monique Kolster, and (D) Barry Rice.

The study was conducted in Merced County within the Central Valley of California on the University of California, Merced Vernal Pool and Grassland Reserve (MVPGR). Merced falls between the hot‐summer Mediterranean and semiarid steppe climate regions of California as described by the Koppen climate types (Kesseli, 1942). The vernal pools are fed by rainfall during the winter; the frequency, amount, and timing of which, combined with the carrying capacity of a pool, dictates the length of the growing season (Zedler, 2003). We studied three observation pools within the MVPGR that were typical of vernal pools on the reserve with respect to depth and size. Along with their average characteristics, they were also chosen due to their ease of access since this is a part of a community science study (Figure 1, Table 1).

**Table 1:** Dimensional and edaphic characteristics of three observation pools on the Merced Vernal Pools and Grassland Reserve. North–south (N‐S) and east–west (E‐W) distances were measured using a transect spanning the pool bottom and 2 m into the pool upland. Pool area was calculated as the elliptical area of the pool. Maximum (max) depth was the height of water in the center of a pool during the wettest year and when the pool was inundated. Estimated volume was calculated using the maximum depth of water and the elliptical area of the pool to determine the hemispheric volume.

Pool	N‐S distance (m)	E‐W distance (m)	Area (m^2^)	Max depth (m)	Estimate volume (m^3^)
1	13.10	21.50	281.65	0.47	34.66
2	13.30	13.70	182.21	0.24	11.45
3	19.10	19.90	380.09	0.31	30.85

The two focal species in this study were whitetip clover and meadowfoam. Meadowfoam is an annual, vernal pool specialist. The cream‐colored flowers are often observed in bloom between early March to late April at the bottom of vernal pools (Hickman, 1993). This meadowfoam species is restricted to hardpan and claypan pools in southeastern San Joaquin Valley (Keeler‐Wolf et al., 1998). The generalist vernal pool associate, whitetip clover, inhabits a wide range of habitats from Alaska to Baja California (Čelakovský, 1874; Hickman, 1993) and can occupy various depths within a vernal pool (Bliss and Zedler, 1998). Whitetip clover is known to flower from March to July across its range (Calflora, https://www.calflora.org). However, to our knowledge its phenological response to climate has not yet been extensively described within vernal pool habitats. We monitored the phenology of both species for 7 years and related phenological variation to within‐year and between‐year precipitation and temperature climate variation. Given the important distinction between growth stages driven by inundation and temperature, we differentiated between early winter (October–December) and late winter (January–March) seasons as done by Javornik and Collinge (2016).

### Climate sampling

Regional precipitation and temperature were derived from measures at the Merced meteorological field station (station ID: 148; coordinates: 37.314, –120.387), managed by the California Irrigation and Management Information System (CIMIS, 2020) approximately 10 km from the study site. Daily measurements of several precipitation and temperature metrics from 1 October 2015 to 30 May 2022 were extracted from the field station: accumulated precipitation, minimum temperature, maximum temperature, and mean temperature. For the early and late winter periods of each year, we calculated the cumulative precipitation, average maximum temperature, average minimum temperature, and average mean temperature.

Growing degree hours (GDH) were calculated as the summation of hours in a day that exceed a temperature threshold. We chose a base temperature of 9.44°C as the threshold because Javornik and Collinge (2016) suggested that vegetation in vernal pool environments commence growth when mean air temperature reaches 9.44°C. Growing degree hours was calculated for early winter (October–December) and late winter (January–March) growing seasons.

Climate moisture index (CMI) for each day was calculated as the total daily precipitation minus the daily potential evapotranspiration + 100 and averaged for the early and late winter seasons to evaluate drought conditions in the vernal pools. Daily potential evapotranspiration was calculated using the Penman–Monteith equation (Monteith, 1965).

Given that the Merced field station was 10 km from the study site, we compared precipitation at the Merced field station to that at the adjacent stations to determine whether the data were representative of regional precipitation patterns. To do this, we compared the accumulated rainfall recorded at four nearby CIMIS climate stations: Denair II, Fresno State, Oakdale, and Orange Cove. The closest and farthest climate stations to Merced were 35 and 128 km away, respectively. A Dunnett's test using the desctools package (Signorell, 2023) in R (v.4.2.0, R Core Team, 2021) was performed to determine whether Merced was significantly different than the four adjacent climate stations.

### Phenology census

Weekly surveys started in late January to monitor the presence of meadowfoam and whitetip clover plants, flowers, and fruits. Once flowers were observed, a team of undergraduate volunteer researchers laid out north–south and east–west transects spanning the length of each pool (Figure 1). Quadrats (10 × 10 cm) were placed every 20 cm along the transect. Flowers within a quadrat were counted once petals had fully unfurled from buds. Flowering onset for each focal species in a given pool was defined as the day when any flowering plant was first observed in that pool, and flowering termination was defined as the date when no flowers were visible in that pool. Flowering duration was the difference between flowering termination and flowering onset. Meadowfoam and whitetip clover plants senesced before 30 May.

### Analyses of phenology responses to climate

We tested for the normality of each phenological variable using the Anderson–Darling statistic with the R package nortest (Zeileis and Hothorn, 2002). The assumption of normality was met for four (meadowfoam and whitetip clover flowering onset and flowering duration) of six dependent variables; flowering termination for meadowfoam and for whitetip clover was non‐normal. To determine whether nonparametric tests were necessary, we first log‐transformed the non‐normally distributed variables. We compared the Akaike information criterion (AIC) value of the models using transformed data and nontransformed phenological measurements as done by Javornik and Collinge (2016). Nontransformed variables performed better in all model scenarios. Thus, we chose to report and assess the results obtained using nontransformed data.

To test for effects of climate variables on flowering onset, termination, and duration, we ran linear multiple regression models in a stepwise fashion for each phenological variable against all climate variables using the lm function in base R, removing nonsignificant predictor variables with the highest *P*‐value until every value was statistically significant (*α* ≤ 0.05). Furthermore, AIC was calculated for each model to determine which performed better. Models with the lowest AIC are reported. All model residuals were tested for patterns of heteroscedasticity using a Breusch–Pagan test using the R package lmtest (Bates et al., 2015).

### Phenological responses to the inundation gradient

To test whether flowering phenology was influenced by the elevation‐based pool inundation gradient, we ran linear regression models of flowering onset, termination, and duration for each quadrat with “distance from edge” as the predictor variable. Observations were taken from quadrats with at least one plant observed. Distance from edge is the distance a given quadrat is from the nearest edge in the pool and can serve as a viable proxy for position along the inundation gradient, which affects both soil moisture and inundation length given that water recedes toward the pool center. Linear regression models were performed using the lm function in base R.

## RESULTS

### Phenological associations with climate

**Figure 2:**
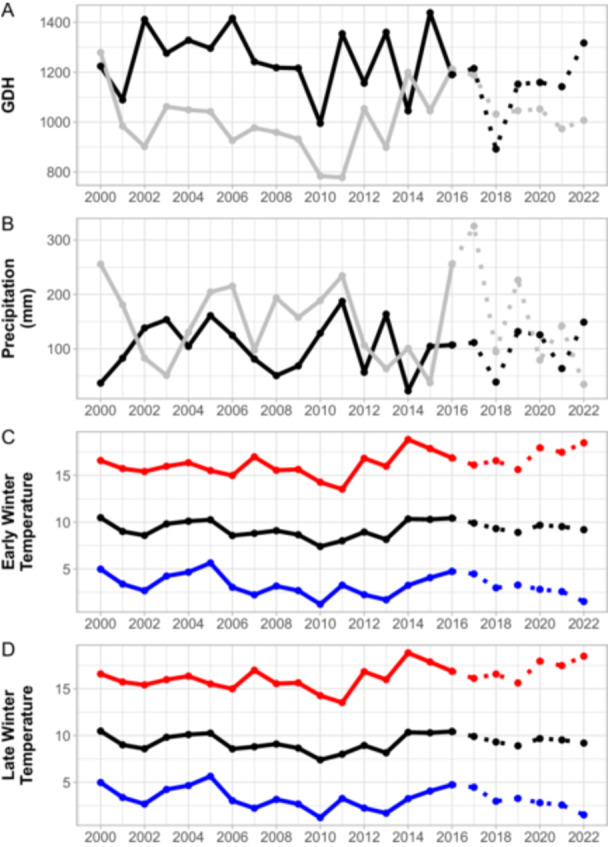
Summary of Merced climate from 2000 to 2022 and during the study period from 2016 to 2022. (A) Growing degree hours (GDH) and (B) volume of rainfall in the early winter season (black line) and late winter season (grey line) with the study period marked by a dotted line. (C) Early winter average temperature and (D) late winter average temperature metrics; daily maximum (red), daily mean (black), daily minimum (blue).

The Merced meteorological station represented a viable regional proxy for climate patterns at the three study pools. An ANOVA test revealed no significant differences among stations regarding accumulated rainfall in either the early or late winter seasons during the study period (early precipitation: *F* = 0.1537, *P* = 0.9598; late precipitation: *F* = 0.1263, *P* = 0.9718; Appendix S1: Figure S1). Winter climate showed significant interannual variability during the study period (Figure 2). Between 2016 and 2022, early winter precipitation fluctuated substantially from a low of 38.8 mm in 2018 to a high of 148.9 mm in 2022 (Table 2), whereas late winter precipitation had a low of 34.6 mm in 2022 and a high of 325.6 mm in 2017. The range of accumulated GDH in early winter was 425°C·h, with a minimum of 892°C·h in 2018 and a maximum of 1317°C·h in 2022 (Table 2). Average early winter and late winter temperature ranged from 10.39°C to 11.40°C and 8.96°C to 10.44°C, respectively. In addition, early winter CMI ranged from 98.35 mm to 99.85 mm over the study period, whereas late winter CMI ranged from 97.96 mm to 101.81 mm, indicating that early winter is generally drier than late winter.

**Table 2:** Summary of the total growing degree hours (GDH), winter temperature, and volume of rainfall for the early and late winter seasons measured during the study period (2016–2022). The day of first rainfall for each year is summarized in the last row.

Year	2016	2017	2018	2019	2020	2021	2022
Early winter GDH (°C·h)	1190.00	1215.00	892.00	1152.00	1159.00	1142.00	1317.00
Late winter GDH (°C·h)	1213.00	1187.00	1032.00	1046.00	1052.00	973.00	1007.00
Early winter mean (C)	11.13	10.85	10.39	10.75	11.16	10.82	11.40
Late winter mean (C)	10.44	9.89	9.35	8.96	9.68	9.52	9.23
Early winter minimum (C)	4.97	4.00	2.61	3.62	3.5	3.33	5.61
Late winter minimum (C)	4.77	4.43	3.07	3.32	2.86	2.58	1.59
Early winter maximum (C)	18.90	19.39	20.50	20.45	20.98	20.90	18.15
Late winter maximum (C)	16.84	16.12	16.55	15.70	17.90	17.46	18.48
Early winter precipitation (mm)	107	111.4	38.8	131.7	125.6	63.7	148.9
Late winter precipitation (mm)	255.6	325.6	94.6	226.4	79.2	142.1	34.6
First rainfall (Julian Date)	274.00	289.00	292.00	275.00	329.00	311.00	294.00

**Figure 3:**
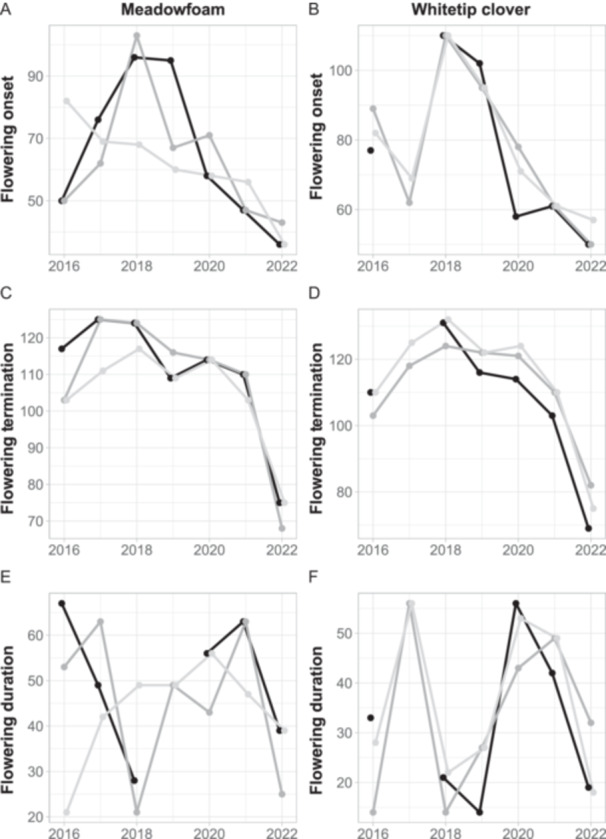
Annual flowering onset (A, B), termination (C, D), and duration (E, F) of meadowfoam (A, C, E) and whitetip clover (B, D, F) from 2016 to 2022. Data are shown for pool 1 (black), pool 2 (dark grey), and pool 3 (light grey).

Flowering onset of meadowfoam and whitetip clover had high interannual variability, with a general trend toward earlier onset and termination during the study (Figure 3). Meadowfoam's date of flowering onset, averaged across three pools, ranged from 89 Julian days in 2018 to 38.3 Julian days in 2022. The date of onset of flowering of whitetip clover ranged from 110 Julian days in 2018 to 52.33 Julian days in 2022. In all years examined, whitetip clover started to flower several days after meadowfoam did. There were moderate but not significant differences between pools in respect to flowering onset, termination, and duration for each species (Appendix S1: Figure S2).

**Figure 4:**
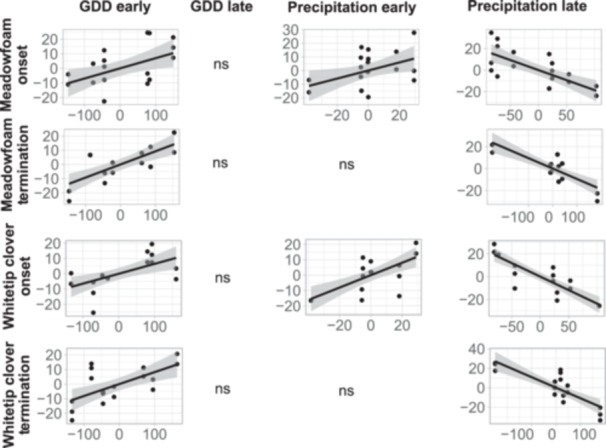
Correlations for flowering onset, termination, and duration with significant predictor variables from stepwise multiple linear modeling. Points represent observations made for one pool in each year. Best‐fit lines (solid line) and standard error (shaded region) are displayed for each significant association.

The phenology of the vernal pool associate and the specialist species showed a clear trend toward earlier flowering onset with warmer and drier winter conditions. Flowering onset and termination of meadowfoam and whitetip clover were negatively correlated with GDH accumulated during the early winter (Figure 4, Table 3). Conversely, GDH during the late winter was not significantly associated with any phenological measurement (i.e., flowering onset, flowering termination, and flowering duration) (Figure 4, Table 3).

**Table 3:** Stepwise multiple linear regression model summaries of early and late winter growing degree hours (GDH) and precipitation as predictor variables of meadowfoam and whitetip clover phenological variables. Observations (Obs.) were made for 3 pools over 7 years. Pool 1 did not host any whitetip clover in 2019, reducing the number of observations to 20.

Phenological variable	Climate predictor	Obs.	Estimates	SE	*P*	*R* ^2^
Meadowfoam onset	Late precipitation	21	0.07	0.03	**0.019**	0.66
	Early precipitation	21	0.30	0.13	**0.041**	
	Late GDH	21			ns	
	Early GDH	21	−0.19	0.04	**<0.0001**	
Meadowfoam termination	Late precipitation	21	0.09	0.02	**<0.0001**	0.74
	Early precipitation	21			ns	
	Late GDH	21			ns	
	Early GDH	21	−0.09	0.02	**<0.0001**	
Whitetip clover onset	Late precipitation	20	0.06	0.02	**0.006**	0.85
	Early precipitation	20	0.40	0.10	**0.001**	
	Late GDH	20			ns	
	Early GDH	20	−0.24	0.03	**<0.0001**	
Whitetip clover termination	Late precipitation	20	0.08	0.02	**0.002**	0.74
	Early precipitation	20			ns	
	Late GDH	20			ns	
	Early GDH	20	−0.11	0.02	**<0.0001**	

Higher precipitation was associated with delayed phenological transitions to flowering for both species. Early winter precipitation was positively associated with flowering onset of meadowfoam and whitetip clover (Figure 4, Table 3). Late winter precipitation was positively correlated with flowering onset and flowering termination of both species (Figure 4).

We found that flowering onset and flowering termination were positively associated with late winter mean temperature and negatively associated with early winter mean temperature (Appendix S2: Table S1). Furthermore, both focal species advanced their flowering onset and termination dates in response to warmer, early winter minimum temperatures. In contrast, warmer, late winter minimum temperatures resulted in delayed flowering termination dates (Appendix S2: Table S1). When maximum temperature was higher in the late winter, flowering start dates of meadowfoam and whitetip clover significantly advanced. The CMI of late winter was positively associated with flowering onset and flowering termination of both focal species (Appendix S2: Table S1).

There was no significant influence of climate—temperature, precipitation, GDH, CMI—on flowering duration for either species. Flowering duration of plant populations, however, varied considerably. Whitetip clover populations were observed flowering for a range of 19 to 56 days averaged across three pools, and meadowfoam populations flowered for a range of 32.7 days to 57.7 days. There was no distinct annual trend toward longer or shorter flowering duration for either species throughout the study period (Figure 3).

### Distance from edge effects on phenology

Meadowfoam and whitetip clover exhibited no change in flowering onset in response to distance from edge, though the termination dates of both species were significantly delayed when individuals were closer to the pool center (Table 4). Meadowfoam exhibited significantly longer flowering durations with greater distance from the pool edge (Table 4; Appendix S1: Figure S3), largely due to consistent flowering onset time and delayed flowering termination. This relationship held for each collection pool and year observed. However, the floral duration of whitetip clover did not contract or lengthen in response to its location in the pool primarily due to high between‐pool variability in respect to flowering onset and termination (Appendix S1: Figure S3). Given that positions closer to the pool center are regions of greater soil moisture and more protracted drying, meadowfoam flowering duration was extended at less‐marginal positions along the inundation gradient.

**Table 4:** Linear regression model summaries of meadowfoam and whitetip clover phenological variables with distance from edge as a predictor variable. Observations (Obs.) are quadrats with at least one meadowfoam or whitetip clover plant collected across three pools and 7 years.

Phenological variable	Obs.	Estimate	SE	*P*	*R* ^2^
Meadowfoam onset	1085	–0.30	0.22	0.169	0.00
Meadowfoam termination	1085	1.07	0.16	**<0.0001**	0.04
Meadowfoam duration	1085	1.37	0.18	**<0.0001**	0.05
Whitetip clover onset	946	0.34	0.22	0.122	0.00
Whitetip clover termination	946	0.37	0.17	**0.033**	0.01
Whitetip clover duration	946	0.02	0.14	0.885	0.00

## DISCUSSION

### Phenological patterns and associations with climate

Meadowfoam, a vernal pool specialist, and whitetip clover, a widespread vernal pool associate, initiated and terminated flowering earlier under warmer and drier conditions in natural vernal pools. Warming promotes earlier germination and growth of vernal pool species (Bliss and Zedler, 1998), and we found that floral phenology also advanced in response to higher annual temperatures for both the vernal pool associate and vernal pool specialist. Laboratory studies of meadowfoam under various temperature conditions revealed faster growth and seed development at higher temperatures, indicating a natural sensitivity to warming (Franz, 1989). While no previous study has examined the effect of warming on whitetip clover, evidence of phenological responsiveness to temperature exists in other clover species such as *Trifolium repens* (Murray et al., 2005), *Trifolium pratense* (Hulme, 2011), and *Trifolium andersonii* (Kopp and Cleland, 2015). These patterns align with those observed in other spring wildflower species that exhibit advanced flowering onset in response to hotter seasonal conditions (Parmesan and Yohe, 2003; Cook et al., 2012; Pearson, 2019).

Our findings indicate that early growth strongly influences phenology later in the life cycle of meadowfoam and whitetip clover, underscoring the importance of monitoring warming trends in both early and late winter. In a study of vernal pools constructed at Travis Air Force Base, California that focused on 11 native and non‐native plant species, Javornik and Collinge (2016) found significant associations of late winter growing degree days (GDD) with plant emergence from 2002 to 2012, but no association with early winter GDD for either native or non‐native species. Although different phenophases were examined (plant emergence versus flowering in our study), this study contrasts with our findings that meadowfoam and whitetip clover phenology responded strongly to early winter GDH. Given that vernal pool plants in natural conditions germinate and grow slowly during the fall and early winter (Keeley and Zedler, 1998), climate during early winter may influence developmental rates. Recent evidence suggests that early growth can play a pivotal role in later floral phenology. *Lasthenia californica* populations that were experimentally seeded in serpentine grasslands in November reached the size threshold for inducing flowering significantly earlier than those seeded in January and March (Olliff‐Yang and Ackerly, 2021). Despite colder temperatures in early winter, variation of interannual temperature is likely to drive differences in vegetative growth rates that lead to advanced or delayed flowering phenology between years as we found in this study.

The delayed flowering transitions observed in 2018, an abnormally cold and dry year in both early and late winter, highlights the importance of considering seasonal conditions that influence all stages of a plant's development. Despite the general trend that dry winter conditions accelerate spring flowering onset, colder temperatures in 2018 may have limited development during the early winter. Accumulated GDH in 2018 was significantly lower than in all other recorded climate years during the study. We hypothesize that poor vegetative growth resulting from low precipitation and cold, biologically restrictive temperatures in 2018 delayed spring flowering onset contrary to phenological expectations under dry conditions. Such delayed flowering, because of delayed germination and vegetative growth, aligns with findings by Olliff‐Yang and Ackerly (2021), although their study occurred in a different habitat type. Monitoring early season vegetative performance would be necessary to confirm our interpretations.

Our findings indicate that inter‐ and intraannual precipitation variability drives shifts in floral phenology of vernal pool species, drawing parallels with some plant habitats while differing from others. Inundation plays a crucial role in wetland plant establishment, germination, and survival (Deil, 2005) and structuring spatial and temporal patterns of community composition in vernal pools (Bauder, 2005; Emery et al., 2009; Javornik and Collinge, 2016). Fall and winter precipitation are primary water sources in vernal pools (Jokerst, 1990), and interannual rainfall variability promotes changes in community composition and phenology (Martin and Lathrop, 1986; Collinge et al., 2013; Javornik and Collinge, 2016). We observed delayed flowering onset and flowering termination in response to higher accumulated rainfall for meadowfoam and whitetip clover. A strong relationship between phenology and hydrology is also found among obligate aquatic plants in ponds and lakes (Calero et al., 2015), where water depth, rather than soil desiccation timing, drives life history transitions of submerged and emergent vegetation. In contrast to aquatic plants, the extent to which changing precipitation patterns drive phenological shifts in terrestrial species remains uncertain across temperate (Sparks et al., 2006), alpine (Hart et al., 2014), Mediterranean (Gordo and Sanz, 2005), and coastal systems (Cleland et al., 2006). However, past research suggests that precipitation plays a clearer role in modulating floral phenology in tropical (Zalamea et al., 2011), subtropical (Peñuelas et al., 2004; Pearson, 2019), and arid regions (Crimmins et al., 2013). Given the ephemeral nature of vernal pools, being both aquatic and terrestrial, it follows that vernal pool plants are likely to respond phenologically to precipitation.

We do not find that climatic variation influences flowering duration for either species. Evidence that flowering duration is less responsive to climate than flowering onset can be found from a community‐scale study on Guernsey island in the English Channel, involving 232 plant species monitored over 27 years, in which 55% of species had significantly earlier flowering onset with warming, while only 19% had a shorter floral duration (Brock et al., 2014). Thus, responsiveness of flowering duration to temperature may be species‐specific though we did find parallels with other studies. Our findings align with those of *Anemone nemorosa*, an herbaceous wildflower, which exhibited a significant earlier flowering onset in response to rising temperatures but no significant shortening of the flowering duration in temperate forests (Puchałka et al., 2022). Conversely, simulated warming shortened the flowering duration of *Arabidopsis helleri* subsp. *gemmifera* (Nagahama et al., 2018) and *Cardamine hirsuta* (Cao et al., 2016) in controlled growth chambers. Shorter flowering durations resulted from delayed floral onset and advanced floral termination for *A. helleri* subsp. *gemmifera* and *C. hirsuta*, respectively. Given that flowering duration is a factor of both flowering onset and termination, two phenophases that may occur earlier or later in response to different exogenous and endogenous factors, a general trend among species may not emerge.

### Phenological patterns of vernal pool specialist and associate

The vernal pool specialist and associate species had similar phenological responses to precipitation, temperature, and drought (Table 3). The regression estimates for flowering onset and termination in response to GDH for both species were nearly identical despite their flowering at different times. Furthermore, nearly all phenological metrics of both species were associated with mean, minimum, and maximum temperatures (Appendix S2: Table S1) despite occupying different regions of the pool and having different biogeographical histories.

Although both species exhibited similar floral responses to climatic variation, there were several differences between the two species' phenological patterns, which may partially explain the differences in their geographic distributions. The mean flowering onset of meadowfoam was 10 days earlier than whitetip clover despite meadowfoam occupying deeper pool zones. Temporal niche segregation is a critical phenomenon for species coexistence (Wiens et al., 1977), particularly in environments with limited space such as vernal pools. In this vein, vernal pool species are present as segregated communities along the pool inundation gradient according to specific adaptations (Schlising and Sander, 1982; Zedler, 1984; Hendrickson, 2024) arising from edaphic and hydrologic differences along the gradual slope of the pool (Holland and Jain, 1984; Zedler, 1984). Whitetip clover appears to be unable to tolerate long inundation periods characteristic of deeper vernal pool zones, which may exclude the species to pool margins. Yet, the floral duration of meadowfoam varied more than whitetip clover with distance to pool edge (a proxy for the inundation gradient). Meadowfoam flowering duration is thus either more sensitive to soil moisture variation, or greater soil moisture variation coincides with the pool zones occupied by meadowfoam. Increased sensitivity to soil moisture variation along the inundation gradient in meadowfoam may partially explain the smaller geographic range of the specialist species, whereas whitetip clover, although less tolerant of inundation, may tolerate a wider range of soil moisture, indicating a possible performance breadth trade‐off (Sexton et al., 2017) between these species.

### Inundation gradient effects on phenology

Plant phenology can be mediated by soil moisture in terrestrial habitats with flowering onset delayed under greater soil moisture (Galen, 2000; Schemske and Bierzychudek, 2001; Dai et al., 2022) or advanced under drought conditions (Franks et al., 2007). Advancing flowering onset and termination under low soil moisture conditions can be advantageous by completing the life cycle before more severe drought occurs (Kooyers, 2015). Alternatively, delaying flowering under high soil moisture can allow for greater biomass accumulation and higher flower production (Dai et al., 2022). Although variation in flowering duration differed by species depending on location in the pools, flowering onset was unassociated with position along the inundation gradient (assessed by distance from edge). These findings contrast with those of other vernal pool plants in which higher soil moisture and delayed soil desiccation dates were associated with later flowering onset among *Lasthenia*, *Downingia*, and *Pogogyne* species (Martin and Lathrop, 1986; Schiller et al., 2000; Collinge et al., 2013). However, we did find a positive association of late winter CMI with time to flowering onset. Furthermore, we found that both meadowfoam and whitetip clover termination dates were delayed in wetter conditions measured both as distance from edge and CMI. Our results add to previous reports that water availability is an important driver of vernal pool floral phenology.

### Implications for climate change

Our results highlight the potential for rapid advancement of mean flowering onset and termination within a relatively short period, consistent with climate change predictions. Over the past 30 years, the climate of the California Central Valley has been trending toward warmer winter and spring seasons (Dettinger and Cayan, 1994; He et al., 2018) with no projected change in precipitation (Stewart et al., 2004; Maurer, 2007). Hydrological models of future climates predict shorter inundation periods for California's vernal pools due to increased warming and evaporation rates (Brooks, 2009; Berghuijs et al., 2014, 2014), potentially endangering vernal pool specialists adapted to longer inundation periods (Bauder, 2005; Brooks, 2009; Montrone et al., 2019). Warmer and drier conditions have led to significant advances of flowering onset among many annual species in various habitats (Abu‐Asab et al., 2001; Primack et al., 2009; Lesica and Kittelson, 2010). In our study, we found that both specialist and vernal pool associate species exhibit profound phenological responsiveness to precipitation and temperature by advancing flowering times under drier and warmer climatic conditions. This suggests that projected climate changes in the Central Valley will likely drive predictable advancements in vernal pool plant phenology. To underscore the potential change in floral phenology, we observed that during the recent 3‐year megadrought in California, meadowfoam initiated flowering in February, a month earlier than reported by Jepson eFlora and a month earlier than all field observations within the Calflora database. These phenological patterns suggest that if warmer and drier conditions become more frequent as projected (Berg and Hall, 2015; He and Gautam, 2016), mean flowering onset is likely to become advanced by a month or more, with unknown ecological consequences. A possible outcome is phenological mismatch between plants and pollinators often reported following phenological shifts in plant species (Miller‐Rushing et al., 2010), potentially reducing population growth rates for plants and pollinators.

Long‐term phenological observations in threatened and vulnerable ecosystems are critical for understanding and responding to global change threats. Our observations indicate that both vernal pool specialists and associates are sensitive and susceptible to potential climate changes in the Central Valley of California. However, plant species from warmer and more variable climates, such as Mediterranean climates, may exhibit higher levels of phenotypic plasticity in various morphological and phenological traits (Kreyling et al., 2019) that could help mitigate or buffer populations somewhat from future climate stresses. We noted high interannual variation in phenology, suggesting the capacity for phenotypic plasticity in meadowfoam and whitetip clover, although further research is needed to confirm significant gene–environment effects. Future research should also examine whether pollinators can track changes in vernal pool plant phenology, providing insights into community‐level consequences of future climate variability.

## CONCLUSIONS

Ephemeral wetlands exhibit high interannual and seasonal variation in inundation, making multiyear studies crucial for understanding community responses to a range of climatic conditions (Pätzig et al., 2020). In this study, we examined vernal pools over 7 years, encompassing climate years with both relatively high and low precipitation compared to the past two decades and wide variation in annual accumulation of growing degree hours. Thus, the time period of the current data set is advantageous for parameterizing the response of natural plant floral phenology to climate.

We found that temperature, precipitation, and the climate moisture index were highly correlated with the phenological timing of meadowfoam and whitetip clover in vernal pool environments. The climate conditions of early and late winter exerted distinct effects on plant phenology, underscoring the importance of capturing broad seasonal variation that influences germination, vegetative growth, and transitions to reproduction. These findings highlight both the inherent variability of vernal pool plant phenology and the significant impact that warmer and drier conditions have on the flowering onset and termination of vernal pool associate and specialist species. Long‐term sampling will be required to observe whether vernal pool plant phenology is significantly advancing in response to ongoing climate change. To our knowledge, this study represents the first examination of climatic drivers of vernal pool plant phenology in the field and offers insights into how phenology may be affected in these vulnerable ecosystems by future climate change.

## AUTHOR CONTRIBUTIONS

B.T.H.: conceptualization, methodology, validation, formal analysis, investigation, data curation, writing original draft, review, editing, visualization, project administration; J.A.B.: conceptualization and investigation; R.M.: conceptualization and investigation; J.S.: conceptualization, methodology, validation, investigation, review and editing, supervision, project administration, and funding acquisition.

## Supporting information


**Appendix S1.** Supplemental figures.
**Figure S1.** Early winter precipitation (A) and late winter precipitation (B) box plots of five CIMIS stations.
**Figure S2.** Box plots of (A) meadowfoam and (B) whitetip clover phenology measures for three observation pools (*x*‐axis) from 2016 to 2022.
**Figure S3.** Meadowfoam and whitetip clover start (blue) and end (red) dates for flowering along the transect of the three pools.

**Appendix S2.** Supplemental table.
**Table S1.** Stepwise multiple linear regression model summaries of (A) mean temperature, (B) minimum temperature, and (C) maximum temperature with precipitation as predictor variables of meadowfoam and whitetip clover phenological variables. (D) Early and late winter climate moisture index (CMI) with precipitation as predictor variables of meadowfoam and whitetip clover phenology.

## Data Availability

The data and code used to produce results for this study are available on github (https://github.com/Brandon-Thomas-Hendrickson/Meadowfoam_WhitetipClover_Phenology.git).
