# Supplementary material for: Climatic determinants of plant phenology in vernal pool habitats
Source: Am J Bot. 2025 Jun 28;112(7):e70064. doi: 10.1002/ajb2.70064 (PMC12281262; doi:10.1002/ajb2.70064)
Supplement: Supplementary file 1 — Appendix S1. Supplemental figures. Figure S1. Early winter precipitation (A) and late winter precipitation (B) box plots of five CIMIS stations. Figure S2. Box plots of (A) meadowfoam and (B) whitetip clover phenology measures for three observation pools (x‐axis) from 2016 to 2022. Figure S3. Meadowfoam and whitetip clover start (blue) and end (red) dates for flowering along the transect of the three pools. [file AJB2-112-e70064-s002.docx]

*P* 0.9286

*P* 0.9997

*P* 0.9749

*P* 0.9996

*P* 0.992

*P* 1.00

Figure S1. Box plots of (A) early winter precipitation and (B) late winter precipitation recorded at five CIMIS stations. The control station, Merced, was contrasted with the four stations that were 35 to 128 km from Merced. Dunnett’s model *P*-values are above the plot for each station that was contrasted with the control.

Hendrickson et al.—American Journal of Botany 2025—Appendix S1

Appendix S1. Supplemental figures.


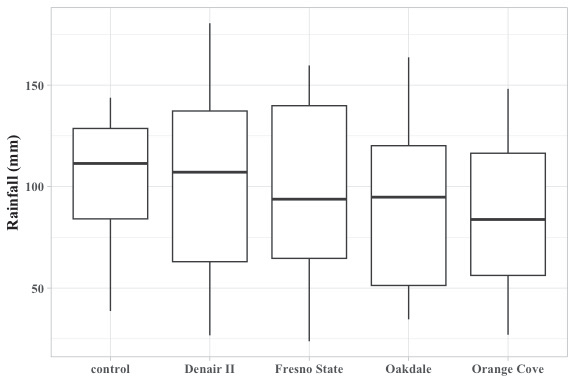

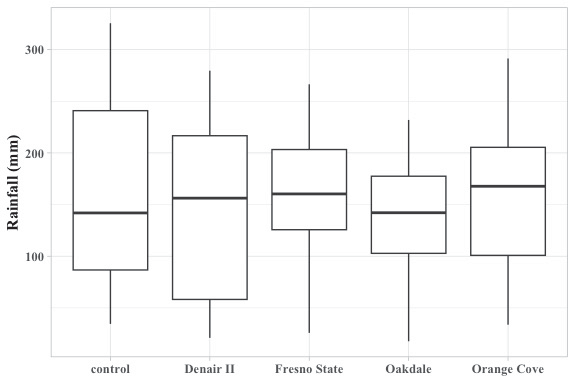


**A**

*P* 0.9731

*P* 0.9205

**B**


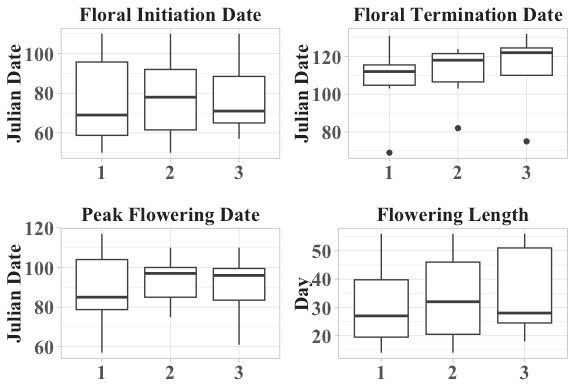

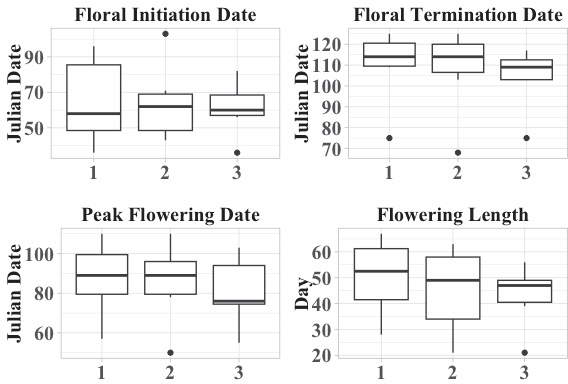


A

B

**Whitetip Clover**

**Meadowfoam**

**Flowering Termination**

**Flowering Termination**

**Flowering Onset**

**Flowering Onset**

Figure S2. Box plots of (A) meadowfoam and (B) whitetip clover phenology measures for three observation pools (*x*-axis) recorded from 2016 to 2022.

**Flowering Duration**

**Peak Flowering**

**Flowering Duration**

**Peak Flowering**


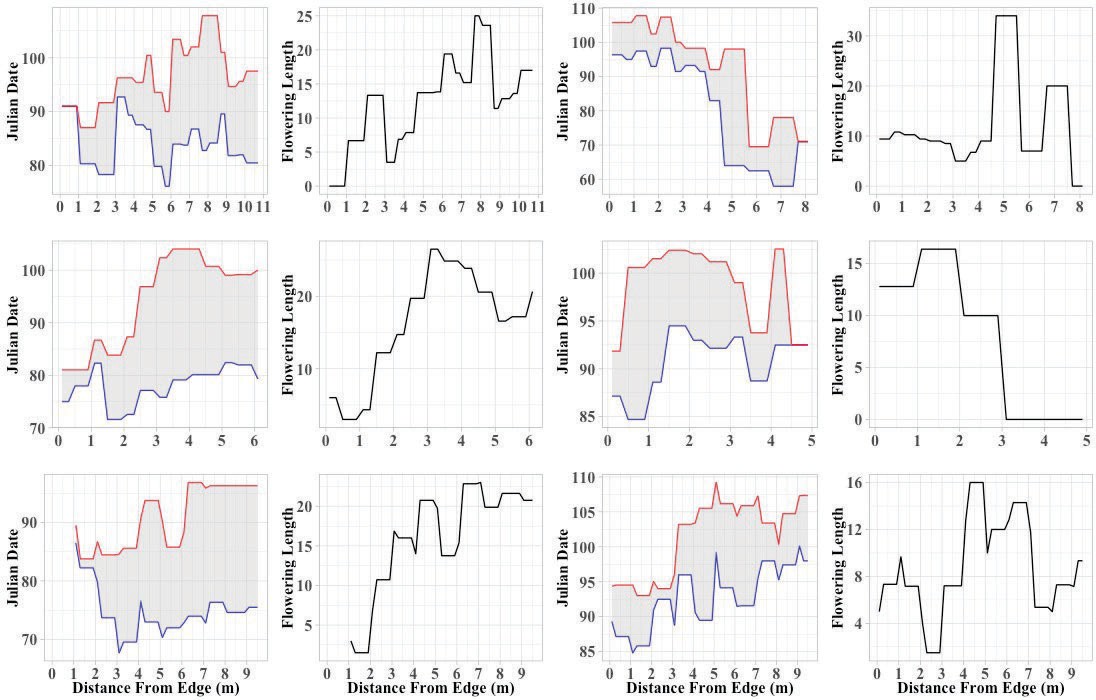


Figure S3. Start (blue) and end (red) dates for flowering of meadowfoam and whitetip clover along the transect of pool 1 (top), pool 2 (middle), and pool 3 (bottom). The shaded region is the flowering duration, which is graphed in the panel on the right for each species.

**Meadowfoam**

**Whitetip Clover**

**Flowering Duration**

**Flowering Duration**

**Flowering Duration**

**Flowering Duration**

**Flowering Duration**

**Flowering Duration**
