# Supplementary material for: Climatic determinants of plant phenology in vernal pool habitats
Source: Am J Bot. 2025 Jun 28;112(7):e70064. doi: 10.1002/ajb2.70064 (PMC12281262; doi:10.1002/ajb2.70064)
Supplement: Supplementary file 2 — Appendix S2. Supplemental table. Table S1. Stepwise multiple linear regression model summaries of (A) mean temperature, (B) minimum temperature, and (C) maximum temperature with precipitation as predictor variables of meadowfoam and whitetip clover phenological variables. (D) Early and late winter climate moisture index (CMI) with precipitation as predictor variables of meadowfoam and whitetip clover phenology. [file AJB2-112-e70064-s001.docx]

Hendrickson et al.-American Journal of Botany 2025-Appendix

Appendix S2. Supplemental table.

Table S1. Stepwise multiple linear regression model summaries of (A) mean temperature, (B) minimum temperature, and (C) maximum temperature with precipitation as predictor variables of meadowfoam and whitetip clover phenological variables. (D) Early and late winter climate moisture index (CMI) with precipitation as predictor variables of meadowfoam and whitetip clover phenology.

A

Meadowfoam Onset

Meadowfoam Termination

SE *P*

103.41 **<0.001**

Meadowfoam Peak

Whitetip Clover Onset

SE *P*

191.46 **<0.001**

Whitetip Clover Termination

SE *P*

150.44 **<0.001**

Whitetip Clover Peak

Predictors Intercept Precipitation Late Precipitation Early Mean Late

Mean Early

Estimates 1052.9

-0.13

0.68

37.31

-127.75

SE 214.13

0.06

0.23

13.36

30.14

*P*

**<0.001**

**0.037**

**0.009**

**0.013**

**0.001**

Estimate

Estimate 825.25

-0.09

0.45

36.56

-102.76

SE 143.02

0.04

0.15

8.92

20.13

*P*

**<0.001**

**0.034**

**0.009**

**0.001**

**<0.001**

Estimate

Estimate

Estimate 1012.07

-0.1

0.64

34.37

-118.99

SE 106.34

0.03

0.11

6.63

14.97

*P*

**<0.001**

**0.004**

**<0.001**

**<0.001**

**<0.001**

585.99

1228.17

-0.16

969.14

0.05

0.2

11.94

26.95

**0.009**

**0.002**

**0.004**

**<0.001**

-0.1

0.56

36.13

-114.08

0.04

0.16

9.38

21.18

**0.035**

**0.003**

**0.002**

**<0.001**

0.21

20.46

-63.64

0.1 **0.046**

4.96 **0.001**

11.62 **<0.001**

0.77

40.23

-145.51

Observations

*R2*

AIC

21

0.672

170.759

21

0.754

156.253

21

0.818

153.808

20

0.779

158.401

20

0.813

148.756

20

0.892

134.881

B

Meadowfoam Onset

Meadowfoam Termination

SE *P*

6.2 **<0.001**

Meadowfoam Peak

Whitetip Clover Onset

SE *P*

16.32 **<0.001**

Whitetip Clover Termination

SE *P*

5.98 **<0.001**

Whitetip Clover Peak

Predictors Intercept Precipitation Late Precipitation Early Minimum Late

Minimum Early

Estimates

109.24

SE

14.62

*P*

**<0.001**

Estimate

Estimate

98.96

SE

11.14

*P*

**<0.001**

Estimate

Estimate

Estimate

107.3

SE

11.78

*P*

**<0.001**

127.14

123.17

139.76

0.11

8.52

-17.11

0.04 **0.017**

1.12 **<0.001**

1.62 **<0.001**

9.05

-12.28

1.12 **<0.001**

1.19 **<0.001**

9.09

-10.98

2.02

2.14

**<0.001**

**<0.001**

7.8

-10.45

2.2

2.25

**0.002**

**<0.001**

-11.63

3.6

**0.004**

11.6

4.02

**0.01**

Observations

*R2*

AIC

21

0.354

178.987

21

0.909

133.44

21

0.731

158.042

20

0.317

175.021

20

0.933

126.17

20

0.68

152.589

C

Meadowfoam Onset

Meadowfoam Termination

SE *P*

Meadowfoam Peak

Whitetip Clover Onset

SE *P*

71.96 **<0.001**

0.04 **<0.001**

Whitetip Clover Termination

Whitetip Clover Peak

Predictors Intercept Precipitation Late Precipitation Early Maximum Late

Maximum Early

Estimates 439.81

-0.1

SE 94.21

0.05

*P*

**<0.001**

**0.044**

Estimate

Estimate

285.55

SE

42.11

*P*

**<0.001**

Estimate

Estimate

90.88

SE

61.19

*P*

0.156

Estimate 445.92

-0.08

SE 61.44

0.03

*P*

**<0.001**

**0.022**

-68.01

0.09

-0.12

45.26 0.151

0.02 **<0.001**

587.5

-0.17

0.06

**0.049**

-0.21

-10.52

0.06

2.53

**0.005**

**0.001**

-21.12

5.17

**0.001**

-28.37

3.94 **<0.001**

-9.93

9.51

2.2

**<0.001**

-20.06

3.37

**<0.001**

8.75

2.09

**0.001**

1.97 **<0.001**

Observations

*R2*

AIC

21

0.513

175.05

21

0.774

154.494

21

0.666

162.614

20

0.763

155.863

20

0.778

148.159

20

0.726

149.545

D

Meadowfoam Onset

Meadowfoam Termination

SE *P*

Meadowfoam Peak

Whitetip Clover Onset

SE *P*

693.09 **<0.001**

1.57 **0.012**

7.2 **0.001**

Whitetip Clover Termination

Whitetip Clover Peak

Predictors Intercept CMI Late CMI Early GDD Late

GDD Early

Estimates

-337.31

5.24

SE 225.1

2.25

*P*

0.151

**0.032**

Estimate

Estimate

-440.81

6.5

SE 138.25

1.38

*P*

Estimate

Estimate

-418.66

6.49

SE 174.7

1.74

*P*

**0.028**

**0.002**

Estimate

-2282.73

6.06

19.75

SE 493.81

1.12

5.13

*P*

**<0.001**

**<0.001**

**0.001**

-502.9

7.14

152.59 **0.004**

1.52 **<0.001**

**0.005** -3072.16

**<0.001**

4.42

30.02

-0.11

0.02

**<0.001**

-0.09

0.02 **<0.001**

-0.11

0.01

**<0.001**

-0.23

0.03 **<0.001**

-0.1

0.02

**<0.001**

-0.16

0.02

**<0.001**

Observations

*R2*

AIC

20

0.886

133.964

21

0.736

155.746

21

0.802

151.602

20

0.859

147.524

20

0.745

150.958

21

0.578

172.075
